# Positive Aspects and Potential Drawbacks of Implementing Digital Teaching/Learning Scenarios in Health Professions Using Nursing Education as an Example: A Research Report from Germany

**DOI:** 10.3390/nursrep14010036

**Published:** 2024-02-22

**Authors:** Lydia Pfeifer, Sophia Fries, Alexander Stirner, Lisa Nagel, Christian Cohnen, Leona Aschentrup, Marleen Schönbeck, Annette Nauerth, Patrizia Raschper, Tim Herzig, Kamil J. Wrona

**Affiliations:** 1 Faculty of Health, Hochschule Bielefeld University of Applied Sciences and Arts, 33619 Bielefeld, Germanylisa.nagel@hsbi.de (L.N.); leona.aschentrup@hsbi.de (L.A.); marleen@schoenbeck.de (M.S.); annette.nauerth@hsbi.de (A.N.); patrizia.raschper@hsbi.de (P.R.); tim_christian.herzig1@hsbi.de (T.H.); 2 Faculty of Educational Science, Bielefeld University, 33615 Bielefeld, Germany; christian.cohnen@uni-bielefeld.de; 3 Faculty of Engineering and Mathematics, Hochschule Bielefeld University of Applied Sciences and Arts, 33619 Bielefeld, Germany; 4 School of Public Health, Bielefeld University, 33615 Bielefeld, Germany

**Keywords:** virtual reality, 360-degree video, digital tools, nursing education, VR-driven scenarios, pedagogical strategies, digital methodologies, immersion, collaboration

## Abstract

Background: Learning arrangements in health care profession education are increasingly taking place in digital environments. Virtual reality (VR) in nursing education, as a digital element, is the subject of controversial debate. On one hand, it supports the authenticity of case studies by adding realistic perspectives and information. On the other hand, the costs of developing and maintaining software and hardware hinder its long-term implementation. Based in the German context, our aim is to promote the adoption of innovative digital methods in nursing education and to offer invaluable experiences from the field. Methods: In this paper, we describe our findings and insights from two different research projects focused on the incorporation of digital tools, particularly VR, into nursing education. Results: Starting with a brief recapitulation of the projects, we elucidate pedagogical strategies for embedding VR-driven scenarios in nursing education. Based on our experiences during the projects, we identify various positive aspects, such as changing perspective and simulating acute situations. Key findings: Although potential drawbacks remain, we advocate the long-term implementation and specific use of VR at the interface between theory and practice. Nevertheless, it is crucial to establish regular evaluation, observing the value of digitalisation, especially VR, for nursing education.

## 1. Introduction

In addition to a shortage of qualified professionals and regional supply shortfalls, current challenges in healthcare professions also include demographic change, which is accompanied by an increasing proportion of chronically ill people and people in need of nursing care [1]. Digital transformation has the potential to address these challenges [2]. It should enable faster and more comprehensive access to high-quality healthcare, uncomplicated communication between service providers and relief for employees in the health sector through the optimisation of processes, such as simple and secure access to health data or digital support for routine activities. In particular, effective relief for employees contributes to raising the attractiveness of the professional field and might counteract the shortage of qualified staff [3]. The changes brought about by digitalisation in the occupational field also have an impact on professional education in healthcare professions, thus demanding curricular integration combined with the educational objective of competent handling of digital media and technologies by health professionals. Additionally, 19 current developments at universities show that, encouraged by online teaching during the pandemic, face-to-face teaching is increasingly complemented by blended learning formats and the use of digital media and technologies [4]. This changing teaching/learning culture is also prominent in education for health professions. Here, too, the use of digital media is increasing. However, their use often does not follow a systematic structure or evaluated pedagogical (subject-related) didactical concepts (ibid). Although digitalisation is integrated into the curricula of health professions in Germany, there is a lack of understanding of digital competence as cross-cutting competence. Against this backdrop, healthcare professionals struggle to actively shape the potential of digitalisation in their work processes [5,6]. To achieve this, educational staff in schools and companies must also be appropriately qualified [7]. Virtual reality (VR) technology can support the aforementioned competence development in the education of health professionals at various points [8]. Due to their design principles, VR glasses offer the phenomenon of immersion and, depending on the design of the experienced scenario, the experience of presence as well [8]. This can reduce visual and acoustic distractions to allow for focused learning in a safe space. Furthermore, a strong experience of presence can evoke emotions in learners that need to be processed and reflected upon in an evaluation discussion or during debriefing in practice or in class. As a result, VR scenarios offer a complement to traditional learning in all learning venues, including the seminar room, simulation environment and practical field [9,10].

Based on the results and experiences of two research and development projects on the topic of the digitalisation of nursing education, this article examines how different forms of VR can be justified in the context of teaching/learning theory and which challenges and possibilities arise with the use of digital teaching/learning scenarios in health professions, using nursing education as an example. This paper is intended to serve both researchers and educational staff in the health sector in the conception of further digitalisation approaches as well as in the design of digital teaching/learning situations in the education of health professionals.

## 2. Theoretical Background

This chapter addresses the current state of research on the topic. This includes both theoretical and empirical findings. However, we do not claim to provide a comprehensive review of the literature on the topic. This chapter, therefore, serves as a brief overview and transition towards our findings. In general, working with problem-oriented case studies is recommended for education in all health professions [11]. Case work and case-based learning are associated with strong practice orientation, increased motivation to learn and the development of analytical, problem-solving and reflective skills [12,13]. Digitally supported case work can enable location-independent, self-directed learning in clinical practice and education. This can also promote interprofessional collaboration between medicine and nursing. If cases are embedded in a learning management system (LMS), further case-relevant, interactively prepared information can be made available in addition to video and audio files. The large amount of additional information is accompanied by greater authenticity of the cases [13]. As a result, in addition to a differentiated view of the case, the computer-supported learning environment promotes in particular the adoption of perspectives and linking with theoretical knowledge [13]. At the same time, the learning medium can bring advantages for both teachers and learners. In addition to increased levels of motivation and engagement [14], learning in the virtual world can help facilitate practical exercises by conserving resources (e.g., material consumption) or repeatedly testing situations that would be costly to recreate in the real world. To achieve this, it is important that what is learned in the virtual exercise is transferred to a practical exercise and thus to a real situation. This is supported on one hand by the realistic interaction with the learning material in VR [15] and on the other hand by the associated addressing of several sensory channels during learning [16]. Additionally, VR can be linked to simulation-based learning, which is often associated with the learning environment skills lab, which is recommended in healthcare profession education and specifically in nursing education [17]. In addition to case-based learning, one aim of simulation-based learning, among others, is the transfer from theoretical knowledge to practical skill during nursing education. VR is often portrayed as an alternative means of simulation which enables more flexibility for the learning process [18].

Against this background, learning arrangements in virtual learning environments are increasingly finding their way into medical, nursing and therapeutic education (or health professions), but at the same time, their development and dissemination is only just beginning [7]. Estimates of the degree of future establishment in nursing education range from short-term hype to a fundamental transformation of nursing education [19,20]. According to the current literature, the following benefits are attributed to the use of VR technology in nursing education [21,22,23,24,25,26]:

- Realistic discussions of rare or dangerous nursing situations;
- Safe training in a virtual learning environment;
- Authentic learning experience with reduced pressure to act;
- Immediate feedback on actions in virtual reality;
- Resource-saving practice with action sequences;
- Learning that is independent of time and place;
- Personalised learning;
- Increased motivation and enjoyment;
- Improved integration of theory and practice;
- The ability to plan the learning experience;
- An improved ability to analyse the learning experience.

Despite these advantages, the nursing and media didactic as well as the learning theory underpinning of the use of VR technology are regarded as controversial [7]. The costs associated with the use of VR technology for the development, acquisition and maintenance of hardware and software, as well as the “not inconsiderable psychophysiological requirements” [23] for learning in virtual learning environments, mean that VR technology currently leads a “shadowy existence” [27]. Additionally, VR is negatively associated with technical problems, cumbersome interfaces and entertainment-driven development [23,27].

In order to minimise the aforementioned costs and development efforts, and to enable educators to create content, an alternative technique can be used instead of the usual creation of digital objects or characters based on geometric modelling: 360° video technology [28]. Such videos are recorded with a 360° camera. These cameras continuously record their entire environment and allow users to view the recording while actively changing their perspective. If several such recordings are available, they can be enriched with interaction icons and linked to a 360° VR scenario. This allows users to interact with people or objects in the scenario and actively change the scenario. It should be noted that here, the range of action is limited. There is no free movement in virtual space, and the options for interaction are more limited than in reality [7]. However, transfer from theory to practice is not yet supported in connection with other learning formats, such as simulation-based learning in the skills lab, for example. This is crucial as the transfer of the VR simulation to reality is of great importance for learning success [13].

## 3. Materials and Methods

Based on the guiding principle that learning with media cannot be ascribed an added value per se compared to conventional learning arrangements but that the media unfold their potential in a coherent didactical design [29], it is important to identify the educational purpose that is being pursued with the use of VR technology. Such an educational purpose may be to promote self-directed and/or cooperative learning, as well as social exchange, or the temporal and spatial flexibility of learning and thus the consideration of heterogeneous learning prerequisites through the use of the digital medium [29,30]. Against this background, the projects “Digital and Virtually Supported Casework in the Health Professions” (DiViFaG) and “Virtual Reality-based Digital Reusable Learning Objects in Nursing Education” (ViRDiPA) tested didactical designs that combine established teaching/learning arrangements with the possibilities of digital and virtual elements.

### 3.1. DiViFaG

The DiViFaG project, funded by the German Federal Ministry of Education and Research (BMBF) in the funding line “Digital Higher Education” (01/2020-12/2022), is an interdisciplinary joint project under the consortium leadership of the Bielefeld University of Applied Sciences and Arts (HSBI) with the participation of the Emden/Leer University of Applied Sciences, Osnabrück University of Applied Sciences and Bielefeld University with its Educational Science and Medicine faculties. The aim of the DiViFaG project is to develop and implement a didactical concept for university education for health professions that combines problem-oriented case studies [31] with innovative human–technology interactions, including virtual reality [32]. For this purpose, the originally text-based case work was enriched with the concepts of cognitive apprenticeship [33] and the learning of social–communicative action competences [32,34].

**Figure 1:**
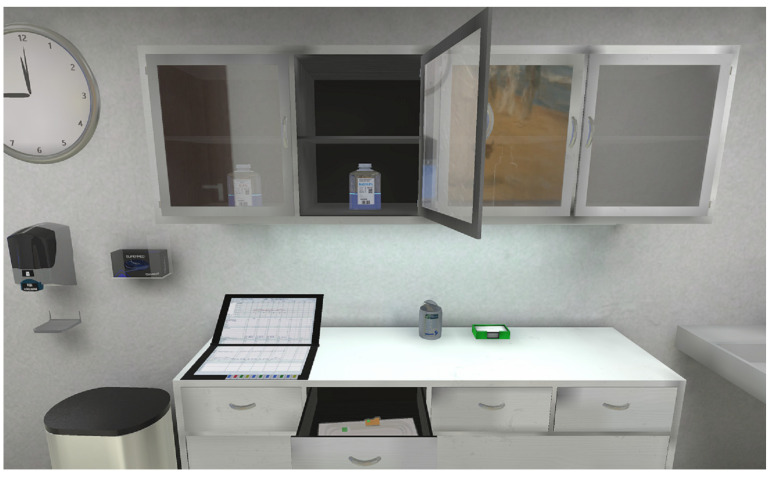
Fully animated ward room from a first-person perspective (DiViFaG).

The project developed, tested and evaluated in total ten case-based, digitally supported teaching/learning scenarios for university education for health professions, in particular medicine and nursing. The case scenarios focus on different practical, communicative and interactive skills. Thus, they strongly address the interface between theoretical learning and practical skills. The digital teaching/learning scenarios were developed as a blended learning concept in which both digital and established teaching/learning methods were linked in classroom and online phases [35]. Due to its immersive character, VR technology was conceptually implemented between the cognitive acquisition of the action steps and the first practical exercise in the skills lab during the presence phase. Through the realistic representation of the action situation (Figure 1), the use of VR has a preparatory effect on learning in the skills lab. The combination of didactical approaches and concepts with digital media and simulative teaching/learning environments facilitates the comprehensive development of students’ competences [32]. All teaching/learning scenarios, including the VR tasks, were evaluated using qualitative and quantitative methods. Approximately 170 students participated in the implementation: 86 of them completed the quantitative online questionnaire, and 31 students participated in qualitative group interviews.

### 3.2. ViRDiPA

Within the framework of the research project ViRDiPA, which was also funded by the BMBF, the University of Bielefeld, the University of Applied Sciences Emden/Leer and an organization with a focus on digital teaching and learning participated in the project period (March 2020–August 2023) under the consortium leadership of the Bielefeld University of Applied Sciences and Arts. An interdisciplinary consortium consisting of actors from nursing didactics, media pedagogy and computer science connected, for the first time, the learning task framework, a proven pedagogical approach in nursing [36], with VR scenarios. Previously analogue learning tasks were digitally prepared and enriched with various media such as images, audio files, videos and tasks. The resulting novel didactical tools enable deep learning by integrating multimodal elements and, in particular, VR applications to promote engagement with the content, thus increasing vividness, situatedness and application orientation. The use of problem-oriented methods such as concrete case studies promotes the cognitive and emotional activation of the learner. In addition, the digital provision of digitally supported learning tasks allows for a flexible organisation of learning in terms of time, space and social interaction. For example, teaching/learning content can be viewed and worked on at home or in the practice facility. In addition, it is possible to work at one’s own pace, which allows for the different needs of learning groups [29].

**Figure 2:**
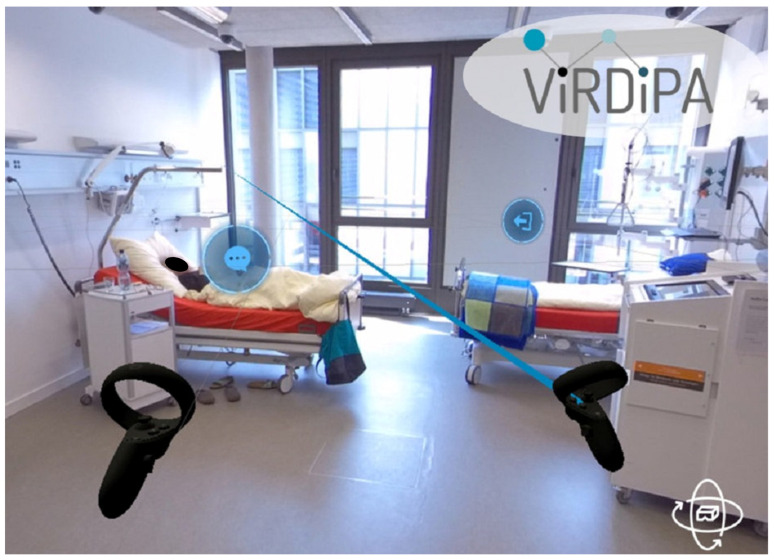
Perceived environment from a first-person perspective with interaction elements (ViRDiPA).

In order to integrate VR technology into education, a training concept was developed, tested and evaluated in order to promote the media-pedagogical competence of educational staff in schools and workplaces with regard to teaching and learning in an immersive learning environment. This educational course enhanced the media and technology skills of 14 teachers and practical instructors. Simultaneously, the participant’s existing subject and nursing pedagogical expertise was combined. In order to transfer learning outcomes (what has been learnt) into everyday working life, the educators developed digitally supported learning tasks with 360° VR scenarios. This particular type of VR scenario is particularly suitable for development by educational staff themselves as no specific programming skills are required. Instead, the authors use the paneoVR authoring kit developed in the project to link 360° videos and enrich them with interaction symbols. See Figure 2 for an example of a 360° VR scenario. The learning tasks and 360° VR scenarios developed in the project were piloted with 114 student nurses. The trials were accompanied by a quantitative and qualitative evaluation, the results of which have been submitted for publication [10]. The development of digitally supported learning tasks with VR scenarios was based on the expectation that they would be highly reusable so that they could be used beyond the context of the project in the cooperating institutions and also be made available to the general public as an open educational resource. Against the background of both projects presented, the didactical designs developed and tested are presented below and analysed with regard to their positive aspects and thus possibilities.

Both projects developed, implemented and evaluated different kinds of VR scenarios and their use in blended learning scenarios in context of linking theory with practice. Both projects published the scenarios and their data as open access material. In addition, the responsible researchers generated various forms of experiences and insights which are delineated in the following chapters. As an example of the use of our VR scenarios in nursing education, specifically in both research projects, a learning unit on dehydration is briefly summarized here. After familiarising themselves with the basics of the subject, learners are presented with a case study relating to dehydration. The learners then reflect on their previous experience of the topic and discuss the options for action in the event of dehydration. As one possible course of action, they practise preparing an infusion in the VR scenario. Then, using a simulated patient, they practise formulating nursing diagnoses, plans and goals. This example shows that VR can only be one building block in a complex learning unit.

## 4. Results: Description of the Major Positive Aspects of Using VR for Education

VR currently presents a special form of learning and teaching. Learning takes place through interaction in recorded nursing situations and creates a feeling of immersion in the care situation. A learning unit in virtual space, therefore, has many potential qualities that can be used in the context of nursing education and can prepare an individual for nursing practice. In both projects, the VR scenarios were treated as additional components to the usual tools in the teaching and learning process. VR was most often used between theoretical and practical learning processes in both methods (case-based scenarios and learning tasks). In general, the students first acquired the theoretical content and discussed possible courses of action in relation to the case example. They then trained the actions in VR in different ways (360° or fully immersive). After that, it was very important to enhance the VR training by practising the actions with a simulated patient in the skills lab or in professional practice. In the following subsections, positive aspects and therefore possibilities are identified as the two projects, ViRDiPA and DiViFaG, are presented. A detailed and broad presentation of the collected data can be found, e.g., in Strecker et al. [37].

### 4.1. Increasing Immersion

VR simulations with a high degree of immersion enable the experience of so-called presence in VR, i.e., the subjective feeling of actually being in the virtual space [8]. Thus, experiencing situations from the perspectives of other actors can feel very real and evoke emotions that are also evoked when actually experiencing the real situation. Thus, by appropriately supplementing subject–didactical methods with animated and 360° VR scenarios, the possibilities for teaching, activating and supporting learners are expanded. According to [29], learning takes place in a situational context, meaning it is linked to social situations. Hence, knowledge acquired in the theoretical setting of an educational institution cannot always be ideally applied in practical situations [29]. However, immersive learning scenarios allow for the provision of targeted learning opportunities in a simulated practice environment in different locations. In addition, immersive scenarios have a high degree of impact due to the potentialities of visual illustration. Constant interaction with the learning content, subjects such as avatars and filmed people and contexts enhance the immersive experience. Application relevance is immediately apparent in immersive learning environments as practice can be carried out in a simulated practical situation. Case descriptions can also help familiarise oneself with patients in advance. To illustrate this, an example of a 360° VR scenario is given below.

In the VR scenario “I didn’t know what to do”, Mrs. Müller’s patient file can be viewed at the nurses’ station. After a little while, Mrs. Müller calls for assistance using the bell system. Upon entering the room, the learner finds Mrs. Müller lying on the floor in front of the bed, moaning and calling for help to get up so she can go to the toilet. The learner now has to decide whether to accompany Mrs. Müller to the toilet or to ask a colleague for help. They are faced with an unexpected situation that requires careful consideration and quick decision making. The confrontation with a helpless person lying on the floor causes consternation, shame and the impulse to help immediately in many learners, but due to the design principles in this scenario, one’s own inability to act in a challenging situation must be endured. The experiences in the 360° VR scenario are prepared and followed up with by reflection questions and discussions within the framework of a learning task. In addition to the VR scenario, the skills lab can be used to practise appropriate actions to take after a fall. For example, an assessment for fractures and serious internal injuries and a transfer from the floor back to the bed can be practised. The vivid and application-oriented representation of learning content makes complex or abstract topics accessible to learners. Direct experience and self-direction contribute to the communication of learning content [38,39]. On one hand, there is a “spatialisation” in which learners have the impression of being in a virtual world. On the other hand, haptic and auditive sensory perceptions are addressed in addition to the visual channels [39]. Learning environments designed according to these principles are actively explored in a self-directed manner whereby new ways of imparting knowledge are created through interaction [40].

### 4.2. Extending Professional Nursing Action through a Change of Perspective

Changing perspectives with other people can be ideally implemented in a 360° VR scenario. A scene can be filmed from the perspective of a fictional person wherein the 360° camera acts as a placeholder for that person. Later, the person’s own perspective can be captured in a 360° VR scenario. This change of perspective, combined with a high degree of immersion, allows important nursing components such as empathy to be trained in interpersonal scenarios. Empathy is an important component of effective communication in the nursing profession and the basis for high-quality, person-centred nursing care. Empathic nursing care has a positive impact not only on the health outcomes of the person being cared for but also on the professional satisfaction of the nurse. One way to promote empathy in learners can be through simulations in which learners take on the role of the person being cared for [41]. Furthermore, changing perspective is not only possible in terms of the client or their relatives but also in terms other professions and actors in the healthcare system. In a multi-player scenario, location-independent collaborative learning is possible in the didactical context of roleplay. For example, an interprofessional ward round or case discussion can be implemented, or interprofessional topics (e.g., hygiene in care) can be integrated into teaching. Communication between learners works directly through the hardware’s voice input and output and can be implemented without additional equipment. The degree of learner self-direction is variable through external teacher intervention or teacher participation in the role play (e.g., as a patient). The high degree of immersion in VR makes both the immersion in the new role and the experience of the situation seem more present and real [37]. On one hand, interprofessionality is a frequently requested building block in the care context to reduce current problems and the duplication of structures; on the other hand, the implementation and permanent integration of interprofessional cooperation in the care context prove to be difficult and challenging. By renewing communication skills and developing interprofessional competences through VR, a further contribution to professional nursing care is made.

### 4.3. Intra- and Interprofessional Collaboration in VR Scenarios

In addition to the initiation of interprofessional competences by adopting the perspectives of other professions in the simulation, collaborative working and learning is possible in VR. Joint learning can initiate shared awareness, mutual trust and respect between health professions and counteract ignorance, prejudice and rivalry [42]. Consequently, interprofessional collaboration and communication, as demanded by experts, is already integrated into education continuously and in addition to existing learning content [43,44]. Interprofessional learning is a prerequisite for later interprofessional work [45]. The central intention of interprofessional learning is to develop the competences required for interprofessional action as part of professional socialisation during education [46]. Interprofessional learning is not about blurring the boundaries between individual professions but rather about working through a common problem by sharing expectations, experiences and perspectives from different roles as part of a collaborative and interactive process [42]. Using roleplay, a treatment plan was developed collaboratively, creating a shared understanding of treatment approaches and perspectives on the same health problem. In principle, collaborative learning in VR can also take place in an intraprofessional context, depending on the objectives of the teaching/learning situation. Here, the focus can be on social–communicative and teamwork skills.

### 4.4. Preparation for Acute Situations

According to Kirkevold (2002) [47], acute situations are characterised by the fact that they occur suddenly, that the situation can take a dramatic course and that quick and correct action is necessary to prevent damage to people’s health. This involves a rapid and appropriate assessment of the situation and a quick decision regarding the immediate action to be taken. In addition, the professional nurse’s own composure and ability to act should be maintained. In addition to working calmly and safely, professional nurses also need to be able to offer their clients an interpretation of their condition and explain interventions and measures [47].

VR scenarios are ideally suited for experiencing acute situations due to several favourable characteristics of the medium. Acute situations are usually difficult to practise in clinical settings because they occur unexpectedly. In a VR scenario, however, all learners can be exposed to a specific simulated acute situation at the push of a button. Experiencing an acute situation in a VR scenario provides a simulated first confrontation with the situation and can allow feelings and emotions to be brought forward into the action-relieved framework of the VR experience. Time for briefing and debriefing can be planned in advance. Although the acute situation is only simulated, VR goggles provide a strong immersion experience [8] so that the learner’s focus is entirely on the simulated acute situation and stimuli from the environment are largely blocked out. Three-hundred-sixty-degree VR scenarios achieve the immersion effect through a filmed environment, while animated VR scenarios use a computer-generated environment. This results in different options for visualisation and interaction. For example, the facial expressions and gestures of filmed persons in 360° VR scenarios can be intuitively recognised, which increases the feeling of authenticity of the situation and thus the affective involvement [10]. With computer-generated avatars, facial expressions and gestures cannot be displayed in the same differentiated way. Animated VR scenarios create additional scope for action or interaction and thus allow necessary steps to be practised in emergency situations in the VR environment.

### 4.5. Observation and Action without Decision-Making Pressure

In VR scenarios, scenes of professional nursing practice can be observed without the pressure of time and action. Due to the realistic depiction of the filmed environment, 360° VR scenarios are ideal for learning by observing. Three-hundred-sixty-degree video recordings can be filmed in an individual practice setting for the familiarisation or recognition of spatial and organisational circumstances. This can increase identification with the content being viewed. Unlike watching videos on a screen, viewing a 360° VR scenario through VR goggles fully immerses the learner in the scenario. The visual and acoustic barrier to the real environment helps avoid distractions. High levels of immersion can be achieved. In the action-free VR environment, the focus can be on observation without the pressure of time and action. Original observation tasks, such as observing an epileptic seizure, are suitable for this, as is the observation of nursing situations, e.g., to reflect the communication of nursing staff. Methodologically, observation-oriented VR scenarios can be prepared for comparison. In this way, learners can watch several similar versions of a scene one after the other and compare them. This also provides the opportunity to observe processes in acute situations (e.g., with experienced nursing staff) without any pressure to act. Animated VR scenarios, on the other hand, are suitable for the repeated practice of concrete, predefined procedures. While in the skills lab, a high level of material consumption is necessary for repetitive exercises, e.g., the preparation of an infusion, learning these action steps in VR has only a low level of resource consumption and is also independent of time and space.

A particular pressure situation arises with action sequences that must be recalled in acute situations. Therefore, for nursing students, this is particularly useful in direct connection with the acquisition of psychomotor skills in the skills lab [40]. With the help of VR scenarios, not only can action situations be practised without time pressure but the learners’ fear of contact can also be reduced through first practical tasks. In the interest of theory–practice transfer, learners are thus prepared for various placements in the healthcare context or are introduced to new areas of application. Typical sounds and minor procedures can be experienced or tried out. Basic nursing measures can be observed, and decisions can be made about the process.

## 5. Discussion: A Critical Reflection on the Use of Digital VR Learning Scenarios in Nursing Education

This section reflects the critical aspects of using VR learning scenarios in nursing education based on the results above by exploring the resource requirements for developing and implementing VR learning scenarios, including costs, hardware and software needs. This document emphasizes the future relevance and reusability of VR scenarios as digital reusable learning objects (DRLOs), accessible as open educational resources (OERs). This section also touches on the challenges of using VR, such as a high initial investment, data protection and the need for continuous updates and adaptations. The suitability of 360° VR scenarios for achieving cognitive and affective learning outcomes is highlighted. In addition, limitations for psychomotor learning are noted. Moreover, the potential for cognitive overload when using VR technology in education is discussed, suggesting the need for structured preparation and follow-up processes.

### 5.1. Resource Requirements for Development, Implementation and Application

In addition to the description of positive aspects of using VR learning scenarios, they must also be critically reflected upon. Regarding the planning and development effort, as well as costs for the procurement of hardware and software, important design principles should be observed when developing a VR scenario. Monetary and personnel costs are considered to be in a reasonable proportion if “future relevance” and “exemplarity” are the guiding didactical design principles in the development of VR scenarios. This means that VR scenarios are developed to address topics which will likely be relevant in the coming years. In addition, the VR scenarios should be suitable for delivering basic learning content in nursing education so that they can be used in different contexts with regard to educational level and teaching topic. Designing such elaborate learning objects should also follow the aim of a “high degree of reusability” [48]. The construction of such reusable digital learning modules with VR technology was the central aim of the projects described here. The VR scenarios developed in the projects were designed as digital reusable learning objects (DRLOs) so that they should be reusable across institutions. They are published as open educational resources (OERs) in an openly accessible form and can thus be used by educational staff at other institutions [7].

As a special form of VR technology, 360° VR scenarios can be produced with less time and money. Even inexperienced teachers can develop these scenarios on their own after a training period, which reduces the resource burden of development. However, when an educational institution decides to use VR technology, the initial investment is high because of the hardware and software that need to be purchased. During the acquisition process, the existing Internet infrastructure must be assessed. A stable and powerful Internet connection is an important prerequisite for the use of VR technology. In addition, especially when learning with VR in health professions, strict data protection regulations must be observed by health institutions when using their internet Infrastructure. Last but not least, the use of VR requires a high level of individual support.

When using VR technology for educational purposes, it is important to remember that it is an innovative technology that is subject to rapid change. Updates from the manufacturers the head-mounted displays require the adaptation of self-developed applications or scenarios to new circumstances. Teaching/learning materials that have already been developed may also lose their relevance and thus their usability.

### 5.2. Three-Hundred-Sixty-Degree VR Scenarios Are Not Suitable for Psychomotor Learning Outcomes

It should be emphasised that 360° VR scenarios can primarily be used to target affective and cognitive learning outcomes. By appealing to the visual and auditory senses, it is possible to evoke emotions which then can be reflected upon during briefing and debriefing. In this way, values and attitudes can be brought to mind and, if the individual so wishes, changed. Cognitive learning outcomes can also justify the use of 360° VR scenarios. For example, knowledge of action sequences or understanding of contexts can be communicated. The key is to combine this with a suitable subject–didactical method that is also geared towards achieving the relevant learning outcomes and allows for comprehensive briefing and debriefing. Due to design principles, a 360° VR scenario can only be viewed from one point of view. There is no freedom of movement in space. Interaction is also limited to the activation of specific interaction icons. Therefore, no motor sequences can be practised. 360° VR scenarios are therefore not suitable for achieving psychomotor learning outcomes. However, it has been shown that the design principles of 360° VR scenarios offer promising possibilities for achieving affective and cognitive learning objectives. Other forms of VR, such as fully immersive scenarios, can be used for psychomotor learning outcomes. It is necessary to include direct perceptible feedback in the VR, e.g., motion tracking, to improve the outcomes of the students.

### 5.3. Cognitive Overload

Students and teachers perceive VR technology as innovative and associate it with entertainment media. This contributes to a high level of attention and motivation when learning with VR. However, enthusiasm for the technology can distract from the subject matter, especially if there is no previous experience with VR technology. Only after a few VR learning sessions and the onset of a habituation effect is it possible to concentrate fully on the learning object. The coordination of the elements used for the scenario is also important. However, a mismatch between the learning and gaming elements is referred to as chocolate-covered broccoli [49].

Although the advantages of VR technology should be used specifically for nursing education, and the attractiveness of VR technology can certainly be used for motivation, it is only by embedding it in subject–didactically designed situations that it is possible to map the nursing process.

In addition, the VR experience should be accompanied by structured, guided preparation and follow-up processes which should also be reflected in the didactically chosen method, such as working with learning tasks. Furthermore, a frustration effect or a certain negative attitude can quickly arise if flawlessness and intuitiveness are not guaranteed. As a result, VR will not promote the desired learning outcomes.

## 6. Conclusions

The didactical use of virtual reality is particularly suitable at the interface between theory and practice. Due to the high degree of immersion, the situation experienced by the learner is close to reality and serves as good preparation for clinical periods in the course of professional education. However, such an application is also conceivable in the context of further education since new fields of action are usually opened up by students in further education. Our report highlights the importance of balancing the innovative potential of VR in nursing education, with its positive aspects and potential drawbacks as listed below (Table 1).

In both projects, the embedding of VR in simulation-based procedures (skills lab) and/or action-oriented teaching procedures (learning tasks and case work) has proved successful. Nevertheless, it is of fundamental importance that the use of VR in the teaching/learning context is accompanied and justified by didactic methods. VR should basically be seen as a digital medium and therefore has an impact on the outcomes, content and methods of a teaching/learning situation. These interdependencies need to be analysed and taken into account. Due to the novelty of VR, a long-term and continuous embedding of this medium in terms of curriculum design seems to be sensible, as it has been shown that learners get used to the degree of immersion and thus the intended learning successes only occur after repeated use. Therefore, it is vital to use VR as well as other digital tools beyond themes and contents of various learning processes to minimize the barriers of VR. In addition, the initiation of digital literacy should be considered, and systematically prepared teachers should be responsible for this. The challenges for appropriate didactics in higher education lie, among other factors, in making digitality the subject of learning processes in studies and education and, in addition, in using digital media—as described here using the example of VR—to design learning situations. It is recognised that digital media serve to support learning and that the actual learning topics will continue to be at the forefront of educational processes. Nonetheless, VR has to be adapted to the intended learning outcomes, the didactical methods, themes and topics as well as the group of learners to integrate it successfully into the curriculum of nursing education. It is crucial to enhance the understanding of digital competence as cross-cutting competence among educational staff and health professionals. 

As already stated at the beginning, the current challenges in the health sector, such as a shortage of skilled workers, regional supply bottlenecks and demographic change, underline the urgency for change and adaptation in the health professions. Digitalisation, especially in the context of nursing education, can be perceived as a promising tool for meeting these challenges. As described, the introduction of VR in nursing education offers an innovative learning tool. However, the following points need to be considered for its successful integration.

There should be greater curricular integration of digitalisation and digital literacy into the education of health professionals. This should go beyond the mere use of digital media, and digital competence should be seen as cross-cutting competence.

Regular evaluation and feedback loops should be established to ensure the effectiveness and added value of digitalisation and the use of VR in education.

To optimise the use of VR and other digital teaching/learning scenarios, universities and other educational institutions should work closely with research institutions.

In the long term, the findings obtained from these projects should be transferred to and implemented in professional practice with a particular focus on the stated positive aspects, as well as potential drawbacks, followed by a scientific evaluation. Altogether, digitalisation and the use of VR in nursing education offer significant opportunities to improve education and address current challenges in the health sector. It is crucial to take advantage of these opportunities and to continuously evaluate and optimise the process.

## Figures and Tables

**Table 1:** Overview of the positive aspects and potential drawbacks of using VR-based scenarios in nursing education.

Positive Aspects	Potential Drawbacks
Resource Efficiency: Less resource-intensive 360° VR scenarios can be developed even by inexperienced teachers. Reusability and Accessibility: VR scenarios are designed as digital reusable learning objects (DRLOs) available as open educational resources (OERs) for widespread use. Educational Impact: Suitable for cognitive and affective learning, VR can enhance knowledge and understanding, evoke emotions and influence attitudes and values.	High Initial Investment: Significant upfront costs for hardware and software. Infrastructure Needs: Requires a stable and powerful internet connection, and compliance with strict data protection regulations. Rapid Technological Changes: Frequent updates and adaptations are necessary due to the evolving nature of VR technology. Limited Psychomotor Learning: 360° VR scenarios are not suitable for psychomotor learning outcomes due to limited interaction and movement capabilities.

## Data Availability

Research data is not yet publically available.

## Public Involvement Statement

The public was involved in the study solely for testing the virtual scenarios. No research was conducted on humans.
